# Splicing Reporter Mice Revealed the Evolutionally Conserved Switching Mechanism of Tissue-Specific Alternative Exon Selection

**DOI:** 10.1371/journal.pone.0010946

**Published:** 2010-06-03

**Authors:** Akihide Takeuchi, Motoyasu Hosokawa, Takayuki Nojima, Masatoshi Hagiwara

**Affiliations:** 1 Department of Functional Genomics, Medical Research Institute, Tokyo Medical and Dental University, Bunkyo-ku, Tokyo, Japan; 2 Laboratory of Gene Expression, School of Biomedical Science, Tokyo Medical and Dental University, Bunkyo-ku, Tokyo, Japan; 3 Department of Anatomy and Developmental Biology, Kyoto University Graduate School of Medicine, Sakyo-ku, Kyoto, Japan; Lehigh University, United States of America

## Abstract

Since alternative splicing of pre-mRNAs is essential for generating tissue-specific diversity in proteome, elucidating its regulatory mechanism is indispensable to understand developmental process or tissue-specific functions. We have been focusing on tissue-specific regulation of mutually exclusive selection of alternative exons because this implies the typical molecular mechanism of alternative splicing regulation and also can be good examples to elicit general rule of “splice code”. So far, mutually exclusive splicing regulation has been explained by the outcome from the balance of multiple regulators that enhance or repress either of alternative exons discretely. However, this “balance” model is open to questions of how to ensure the selection of only one appropriate exon out of several candidates and how to switch them. To answer these questions, we generated an original bichromatic fluorescent splicing reporter system for mammals using fibroblast growth factor-receptor 2 (FGFR2) gene as model. By using this splicing reporter, we demonstrated that FGFR2 gene is regulated by the “switch-like” mechanism, in which key regulators modify the ordered splice-site recognition of two mutually exclusive exons, eventually ensure single exon selection and their distinct switching. Also this finding elucidated the evolutionally conserved “splice code,” in which combination of tissue-specific and broadly expressed RNA binding proteins regulate alternative splicing of specific gene in a tissue-specific manner. These findings provide the significant cue to understand how a number of spliced genes are regulated in various tissue-specific manners by a limited number of regulators, eventually to understand developmental process or tissue-specific functions.

## Introduction

Genome projects have shown that metazoans generate a hugely diverse proteome from a limited number of genes. This finding underscores the importance of alternative splicing, through which a single gene can generate multiple structurally and functionally distinct protein isoforms. Moreover, recent transcriptome analyses with splicing-sensitive microarrays or deep sequencers have revealed that alternative splicing occurs in more than 90% of multi-exon genes in human [1] and over 60% of these cases are regulated in a tissue- and cell type-specific manner [2]. Alternative splicing is regulated by auxiliary cis-elements with regulatory proteins that enhance or repress splicing of adjacent exons [3], [4] however, the mechanism by which a number of genes are regulated in various tissue-specific manner by a limited number of regulatory factors remains unclear.

In mammals, fibroblast growth factor-receptor 2 (FGFR2) is one of the best characterized gene in which mutually exclusive alternative splicing produces two isoforms. Exon 8 (also termed IIIb) isoform is specifically expressed in epithelial tissues, whereas exon 9 (or IIIc) isoform is selected in non-epithelial or mesenchymal tissues [5], [6]. The structural difference between two splice isoforms markedly affects the specificity of ligand–receptor binding [7], [8], [9], and exon switching is shown to be essential for development in the mouse [10], [11]. Several factors have been identified which positively or negatively regulate either of alternative exons of FGFR2 independently. For exon 8 regulation, Del Gatto-Konczak et al. found that heterogeneous nuclear ribonucleoprotein, hnRNP A1, binds to exon 8 (also termed K-SAM exon) as ESS (exonic splicing silencer) and represses its inclusion [12]. Carstens et al. found the polypyrimidine tract binding protein (PTB) represses exon 8 inclusion through ISS-1 and ISS-2 (intronic splicing silencers-1 and 2) [13]. Warzecha et al. recently cloned RBM35a and RBM35b as epithelia-specific activators of exon 8 inclusion, and renamed them epithelial splicing regulatory proteins 1 and 2 (ESRP1 and ESPR2), respectively [14]. For exon 9 regualtion, Chen et al. found that Tra2β represses the selection of exon 9 [15]. Baraniak et al. reported that Fox2 represses selection of exon 9 through binding to a UGCAUG sequence in intron 8 [16]. Hovhannisyan et al. found that a hnRNP M binds to ISS-3 and represses inclusion of exon 9 [17]. Mauger et al. showed that hnRNP H and F interact with Fox2 and repress exon 9 inclusion [18]. Also, presence of unknown enhancer is speculated for exon 9 inclusion through ISE (intronic splicing enhancer) in intron 9 [14]. So far, mutually exclusive splicing regulation is widely believed as the outcome from the balance of multiple regulators that enhance or repress either of alternative exons discretely [19], [20]. However, this “balance” model is open to questions, 1) How the balance of multiple regulators can ensures the selection of only one appropriate exon out of several candidates? 2) Whether transcriptional or post-transcriptional control of multiple regulators is possible to achieve distinct switching of alternative exons? 3) If 2) is the case, how to control the multiple regulators at once for exon switching? To answer these questions, we generated a bichromatic fluorescent splicing reporter system for mammals using FGFR2 gene as a model. This reporter contains entire cis-elements necessary to reproduce native alternative splicing regulation and enable us to visualize and monitor it *in vitro* and *in vivo*. The transgenic mice expressing this splicing reporter clearly showed the epithelial tissue-specific splicing pattern throughout their entire bodies. By using this splicing reporter, we demonstrate that key regulators define the single exon expression in the tissue-dependent manner through the ordered splice-site recognition of the mutually exclusive exons.

## Results

### Generation of the FGFR2 splicing reporter system

**Figure 1:**
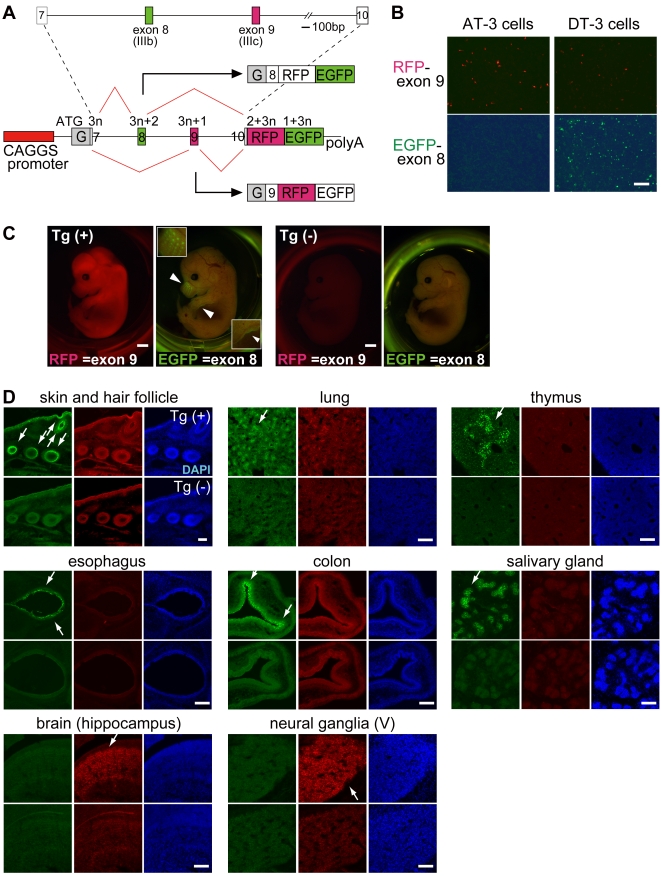
Construction of FGFR2 splicing reporter vector and their expression patterns. (A) Scheme of FGFR2 splicing reporter vector. The genomic fragment of mouse FGFR2 including exon 7 through 10 was amplified and introduced into the reporter vector containing a CAGGS promoter and RFP-EGFP with different reading frames. Modified glutathione-S-transferase gene (indicated as “G”) was inserted in front of the exon 7 in-frame. A schematic representation of the mRNA derived from the reporter under the alternative splicing regulation is also shown; the numbers indicate the reading frames. (B) Expression pattern of splicing reporter in vitro. The reporter vector was introduced into two rat prostate cancer cell lines AT-3 and DT-3, which have different cell-type specificities. Scale = 200 µm. (C) Expression pattern of splicing reporter in vivo. Fluorescence images of transgenic reporter mouse embryos at E14.5. Tg(+) is an embryo carrying the reporter vector, and Tg(–) is one of its litter-mate lacking the vector. Arrowheads in Tg(+) indicate EGFP signals with the patterns of whiskers (upper arrowhead) and the edge of a limb (lower arrowhead), both of which are magnified and indicated by white rectangles in the upper left-hand and lower right-hand corners, respectively (scale = 1 mm). (D) Sections from transgenic reporter mouse embryos at E16.5. Each panel shows sections from the indicated tissues, the upper one from Tg(+) and the lower from Tg(–). Portions expressing the EGFP signal are indicated by white arrows (scale = 100 µm).

We used a 3.7-kb genomic fragment of FGFR2 gene that included two alternative exons (exon 8 and 9) flanked by their upstream and downstream exons (exon 7 and 10), with introns in between (Figure 1A). By using mostly entire genomic region around alternative exons, this reporter system was expected to contain all the regulatory cis-elements essential for tissue-specific regulation and to tell which splice site sequences and cis-elements are truly critical for regulations. The genomic fragment was cloned into a vector containing RFP and EGFP in tandem with different reading frames [21]. With this reporter system, splicing regulation could be monitored from a single reporter vector that expresses either EGFP when exon 8 is chosen or RFP when exon 9 is selected (Figure 1A).

By using two prostate carcinoma cell lines, AT-3 and DT-3 cells, we then examined whether the reporter system could reflect cell type-specific splicing regulation. AT-3 cell is a mesenchymal-type cell that specifically expresses exon 9 isoform of endogenous FGFR2, and DT-3 cell is an epithelial-type cell that predominantly expresses exon 8 isoform [22]. When this reporter system was introduced into these two cell lines, AT-3 cell specifically expressed exon 9-RFP and DT-3 cell predominantly expressed exon 8-EGFP (Figure 1B). We therefore could confirm that our reporter vector reflects cell type-specific regulation of endogenous FGFR2 splicing.

We next checked whether our reporter system could show tissue-specific regulation of FGFR2 splicing in vivo by generating transgenic mice from this reporter. A well-known change in the FGFR2 splicing isoforms occurs in mouse development stages from E14.5 to E16.5. In these stages, differentiation of future epithelial cells is induced and they start expressing exon 8 isoform of FGFR2 to receive morphogen signals, such as FGF-10, from mesenchymal cells [23]. When the whole body of a transgenic embryo was examined at E14.5, a broad RFP signal was detected throughout the entire body, and a specific EGFP signal was detected as the whisker pattern and on the edges of limbs or the body (Figure 1C). We further evaluated the detailed expression profile through examining series of sections from the transgenic embryos in the late development stage of E16.5 (Figure 1D). An EGFP signal was detected specifically in cells on the surface of the skin and bulbs of hair follicles, where differentiated epithelial cells were located (Figure 1D, shown with arrows). Also, the EGFP signal was detected at epithelial cells in the alveoli of the lung, in the esophagus and colon, at the thymus epithelia, and at the salivary gland ductal cells (Figure 1D, arrows in the top and middle panels). A strong RFP signal was detected in the developing brain (hippocampus) and peripheral nervous system (trigeminal ganglia) (Figure 1D, arrows in bottom panels). Expression patterns of EGFP were compatible with reported FGFR2 exon 8 expression patterns [23], [24], indicating that our reporter system reflects endogenous splicing regulation of FGFR2 in vivo and the genomic fragment used in the vector contains the regulatory elements necessary for tissue-specific switching of mutually exclusive exons.

### Unbalanced sequence of 3′ splice site is essential for mutually exclusive exon selection

**Figure 2:**
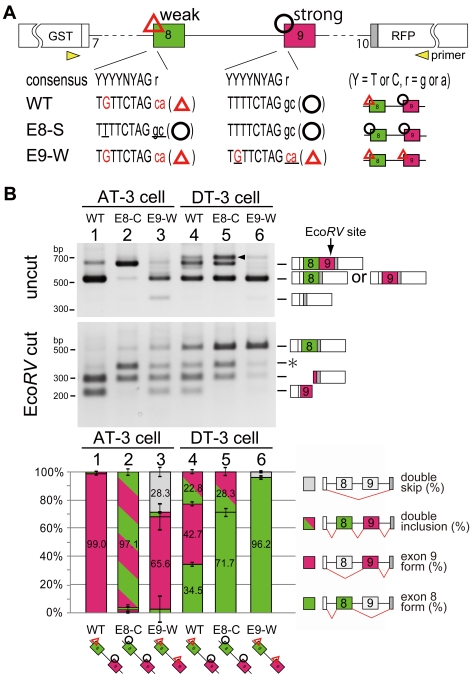
Unbalanced sequence of 3′ splice sites is essential for mutually exclusive exon selection. (A) Scheme for 3' splice site mutation on exons 8 and 9. Uppercase letter is intron and lowercase is exon sequence. Red characters indicate mismatches from the conserved consensus sequence in the 3' splice site and poly-pyrimidine moiety, and underline indicate mutated sequence. The yellow arrows with “primer” represent the positions amplified in RT-PCR. (B) RT-PCR from AT-3 and DT-3 cells transfected the indicated vectors. Splice products were digested with Eco*RV*, which uniquely cuts the PCR product containing exon 9. Each band was identified and indicated with the scheme of splice products. Arrowheads indicate nonspecific PCR products, which was confirmed by sequencing. The asterisk indicates the splice product came from double inclusion of exon 8 and exon 9. The bar graph shows the amount of each splicing product, and is based on calculations from three independent experiments; the mean value for each splice product is show in the respective column with an error bar showing the SD (standard error).

In the embryos of splicing reporter transgenic mouse, RFP was expressed almost throughout the entire body, and EGFP was specifically expressed in epithelial cells. This expression pattern suggested the possible regulatory mechanism that exon 9 was dominantly selected as “default” in reporter transgenic mouse, and epithelial-specific regulators might promote inclusion of exon 8. To test this hypothesis, we initially compared sequence of alternative exons including their 3′ and 5′ splice sites. The major difference identified between these two exons is that exon 8 has a weaker 3' splice site and a polypyrimidine moiety that contains several mismatches from the consensus sequence (Figure 2A, TGTTCTAG ca), whereas exon 9 has stronger 3' splice site which has conserved consensus sequences (Figure 2A, TTTTCTAG gc). There are no obvious differences in their 5' splice sites (data not shown). To examine whether the unbalanced 3' splice site is essential for “default” selection of exon 9 in non-epithelial cells, we introduced mutations in their 3' splice sites and observed change in splicing regulations. We prepared two types of mutated vectors, one has the same stronger 3' splice sites on both exon 8 and 9 (Figure 2A, E8-S vector), and the other has the same weaker 3' splice sites on both exon 8 and 9 (Figure 2A, E9-W vector). These vectors were transfected into AT-3 and DT-3 cells, and the change of splicing regulation was examined by RT-PCR. When WT vector was introduced into AT-3 cell, it adopted almost 100% of the exon 9 form, whereas DT-3 cell adopted around 45% {34.5/(34.5+42.7)%} of exon 8 form among the single inclusion product (Figure 2B, lane 1, 4), which was consistent with the expression pattern of fluorescence in Figure 1B. Our splicing reporter was designed not to cause early premature termination codon in double-inclusion form to escape the nonsense-mediated decay (NMD) reaction [25]. We therefore could monitor all splicing products, including the double-inclusion and the double-skip forms. Strikingly, when E8-S vectors were transfected, AT-3 cell mostly expressed the double-inclusion form, meaning that two alternative exons were processed as constitutive exons (Figure 2B, lane 2). This result indicates that the weaker 3′ splice site of exon 8 is critical for single exon selection of exon 9 from two mutually exclusive exons in non-epithelial AT-3 cell (Figure 2B, lane 2), though the ESS or ISS on or close to exon 8 may be required for the complete suppression [13], [26], [27]. When the 3' splice site of exon 9 was weakened (E9-W vector), selection in DT-3 cell almost fully switched to exon 8 (Figure 2B, lane 4 and 6), indicating that full repression of exon 9 might be important for exon 8 inclusion in epithelial DT-3 cell. Thus unbalanced 3' splice sites are essential for the single exon choice from mutually exclusive exons and for their switching.

### Disruption of exon 9 causes switching to exon 8

**Figure 3:**
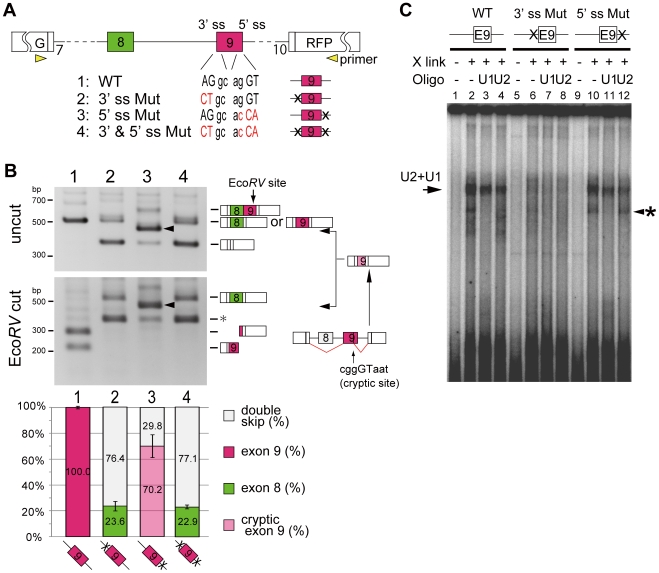
Promoted selection of exon 8 by disruption of 3′ splice site of exon 9. (A) Scheme of 3' and 5' splice site mutation on exon 9. Uppercase letter is intron and lowercase is exon sequence, red characters indicates mutated sequence. (B) RT-PCR from AT-3 cells into which the indicated vectors were introduced. Arrowshead indicate aberrant spliced product that used the 5' cryptic splice site inside exon 9. The bar graph, which represents the amount of each splicing product, is based on calculations from three independent experiments; the mean value for each splice product is show in the respective column with an error bar showing the SD (standard error). (C) Results of the in vitro splice site recognition assay. The scheme for exon 9 shows the position of the splice site mutation as “x”. “X-link” shows the presence or absence of UV-induced crosslinks in samples after the in vitro splicing reaction. “U1 oligo” and “U2 oligo” represent the digestion of RNA samples by RNaseH with complementary oligos for U1 or U2. The band shown by arrowheads with asterisk may be a probe crosslinked with U1 that binds to the cryptic 5' splice site inside exon 9, because it was detected in the 5' ss mutated probe and digested with U1 oligo.

Results in Figure 2 showed that weaker 3' splice sites of exon 8 is essential for the single exon selection of exon 9 in non-epithelial AT-3 cell. And epithelial DT-3 cell efficiently chose exon 8 form when 3′ splice site of exon 9 was weakened. These observations suggest a possibility that repression of exon 9 causes switching to exon 8. To examine this hypothesis, we introduced mutations on either or both of the 3' and 5' splice sites of exon 9 to destroy its splice site consensus sequence mimicking repression, and transfected these into AT-3 cell (Figure 3A). When both splice sites of exon 9 were mutated (Figure 3A, 3′&5′ ss Mut), AT-3 cell expressed the exon 8 form (22.9%) and the double-skip form (77.1%) (Figure 3B, lane 4). These results indicated that blocking of exon 9, at least partially, promotes switching to exon 8 in AT-3 cell. Interestingly, mutation of the 3' splice site (Figure 3A, 3′ ss Mut) was just sufficient to cause this switching (Figure 3B, lane 2), whereas mutation of the 5' splice site (Figure 3A, 5′ ss Mut) produced an aberrant splicing product of exon 9 using a cryptic 5' splice site at ggGT in exon 9 (Figure 3B, lane 3 indicated by arrowhead and scheme was illustrated on the right side). These results indicate that recognition of 3′ splice site is essential for exon 9 selection, suggesting the possibility that recognition of exon 9 is its 3′ splice site dependent. To test this hypothesis, we performed in vitro splicing assay to directly monitor the splice site recognition by U2 and U1 snRNA/snRNP binding (Figure 3C). The ^32^P-labeled RNA probes for wild-type and mutated exon 9 containing the flanking introns (top panels of Figure 3C) were crosslinked by UV irradiation after incubation with HeLa nuclear extract and separated by electrophoresis. HeLa cell was confirmed to have non-epithelial cell character. The specificity of U2 or U1 binding was confirmed by addition of an oligonucleotide complementary to U2 or U1, and RNase H digestion [28], [29] (Figure 3C and Figure S1). In the splicing conditions, binding and shift of U1 and U2 snRNAs were observed with the WT RNA probe, in which the U1 and U2 bands overlap (Figure 3C, lane 1–4, and Figure S1, lane 1–4, indicated by an arrow). They became fainter by RNase H digestion with U1 (Figure 3C, lane 3, and Figure S1, lane 3) or U2 oligos (Figure 3C, lane 4, and Figure S1, lane 4). Shifted band almost disappeared with double digestion with U1+U2 oligos (Figure S1, lane 7), while the band was resistant against the RNase H digestion with U6 oligo (Figure S1, lane 8), indicating that the exon 9 RNA probe is recognized by U1 and U2 oligos in this splicing condition. Strikingly, the 3' splice site mutation of the exon 9 RNA probe resulted in a significant loss of the shifted band (Figure 3C, lane 6–8). These results suggested that recognition of exon 9 primary depends on the binding of U2 snRNA to the 3' splice site. On the contrary, with the probe harboring with 5' splice site mutation (5' ss mutation), binding of both U1 and U2 was retained (Figure 3C, lane 10–12), in good accordance with the results of RT-PCR shown in Figure 3B, lane 3. These results suggest a possibility that binding of U2 snRNA supports the binding of U1 snRNA, so that much weaker cryptic 5' splice sites in exon 9 was used in its 5′ ss mutation (Figure 3B, lane 3 and Figure 3C, lane 12 indicated by an arrowhead with an asterisk). These observations give a possible explanation why the selective use of exon 9 in non-epithelial cells depends on the relative strength of its 3' splice site.

### Identification of silencing elements for exon 9 recognition

**Figure 4:**
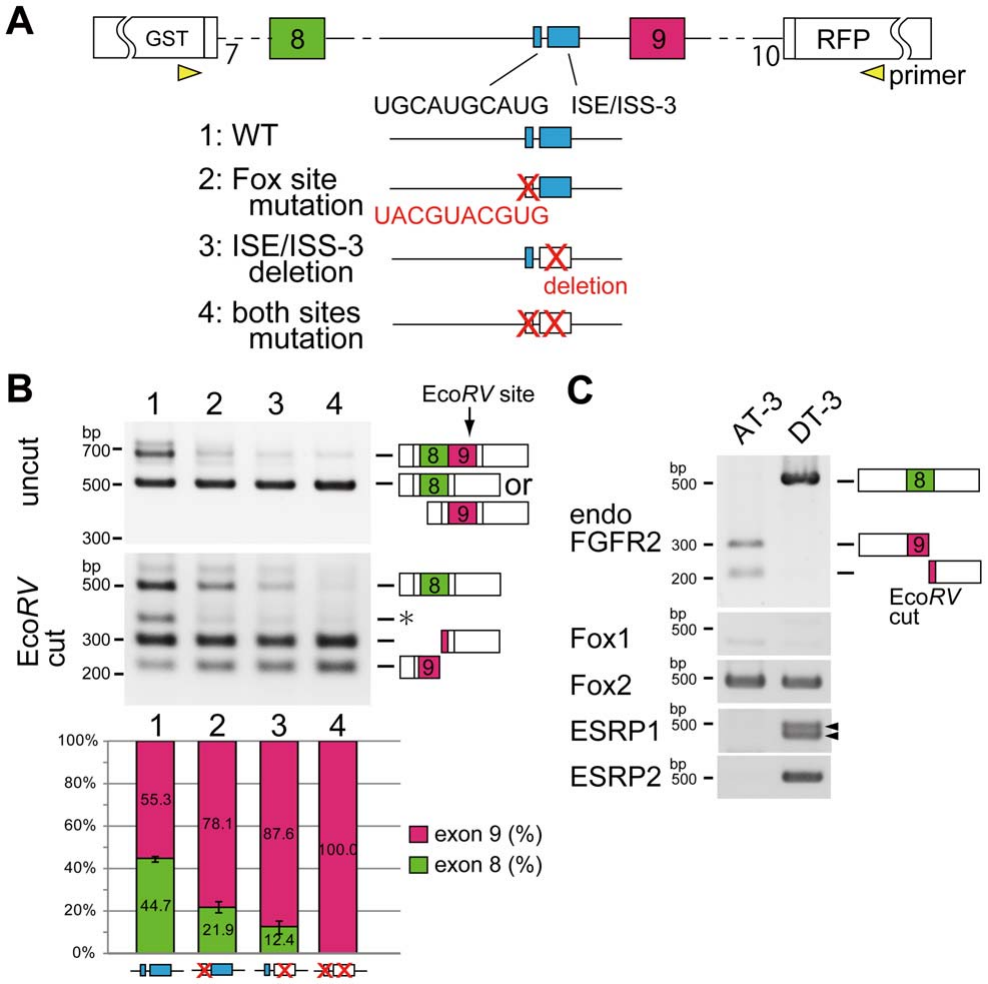
Identification of silencing elements for exon 9 recognition. (A) Scheme of cis-mutation experiment on UGCAUG and ISE/ISS-3 which is located upstream of exon 9. Red characters indicated mutated sequences or deletions. (B) RT-PCR from AT-3 cell into which the indicated vectors were introduced. The bar graph shows the amount of each splicing product, and is based on calculations from three independent experiments; the mean value for each splice product is show in the respective column, with an error bar showing the SD (standard error). (C) RT-PCR from AT-3 and DT-3 cells showing amplified endogenous FGFR2, Fox1, Fox2, ESRP1, and ESRP2. The arrowhead in ESRP1 corresponds to two splice isoforms which was confirmed by sequencing.

Non-Epithelial or Mesenchymal regulation, unbalanced 3' splice sites are essential for single exon selection of exon 9 in non-epithelial cells and recognition of exon 9 is its 3′ splice site dependent. Also disruption of this 3' splice site of exon 9 partially caused switching to exon 8. These results suggest the presence of silencer(s) for exon 9 to cause switching to exon 8 in epithelial cell. To test this hypothesis, we initially screened suppressive cis-elements located near the 3' splice site of exon 9, and picked up two highly conserved sequences: the UGCAUG sequence and ISE/ISS-3 (intronic splicing enhancer/silencer-3) in intron 8, both of which have been reported as the silencing cis-elements for exon 9 [16], [30] (Figure 4A). To examine whether these two cis-elements are essential for silencing exon 9, we introduced mutations in either or both UGCAUG and ISE/ISS-3 in our reporter, and transfected into epithelial DT-3 cell. First, we substituted UGCAUGCAUG for UACGUACGUG to disrupt the binding to the RNA-binding protein of Fox, which was reported as the repressor of exon 9 [16]. Then, the ratio of exon 8 selection in DT-3 cell fell by a half (44.7% to 21.9%, Figure 4B, lane 2). Next, we deleted ISE/ISS-3, an 85-bp sequence containing several dinucleotide GU sequences. The deletion of ISE/ISS-3 reduced the ratio of exon 8 inclusion to one-fourth (44.7% to 12.4%, Figure 4B, lane 3). When both of these elements were mutated, DT-3 cell could no longer choose exon 8, and all splicing products were the exon 9 form (Figure 4B, lane 4). These results indicate that DT-3 cell use both of these cis-elements to select exon 8 presumably by silencing the exon 9 via its 3' splice site.

### Regulatory mechanism of transacting factors to switch exons

**Figure 5:**
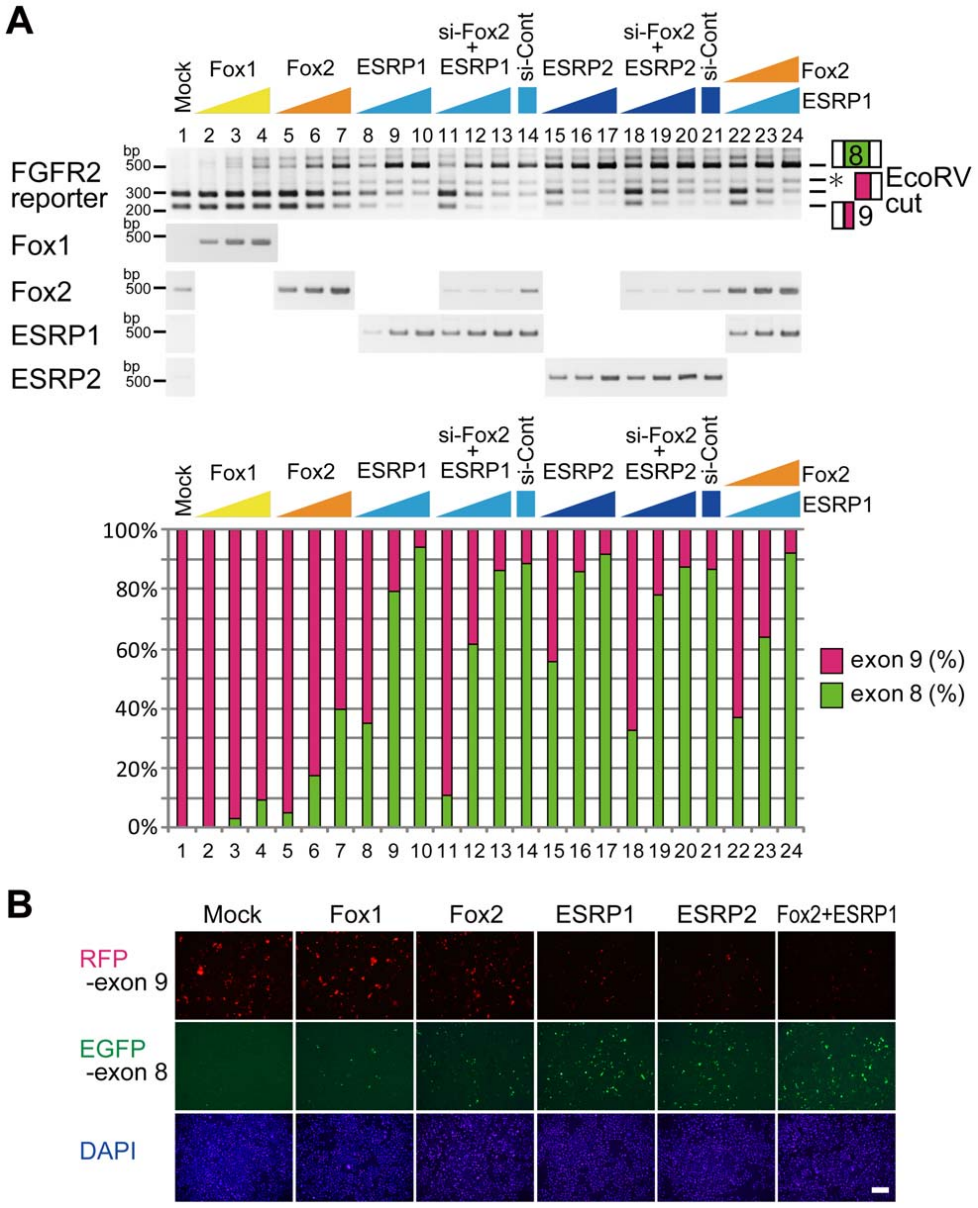
Foxs and ESRPs promote switching from exon 9 to exon 8. (A)RT-PCR from HeLa cell transfected the wild-type reporter and indicated cDNA expression vectors with or without Fox2 siRNA. The bar graph shows the amount of each splicing product. (B) Fluorescent microscopy image of HeLa cell transfected the wild-type reporter with indicated cDNA expression vectors (scale bar = 200 µm).

Results from Figure 4 suggest that both UGCAUG and ISE/ISS-3 are necessary and sufficient for selecting exon 8. A previous study has shown that Fox2 promotes exon 8 inclusion through UGCAUG in intron 8 [16]. Also, a recent study from cDNA library screening identified epithelial splicing regulatory protein ESRP1 and ESRP2, which mediate exon 8 inclusion through binding to ISE/ISS-3 [14]. We therefore examined whether Fox1, Fox2, ESRP1, and ESRP2 promote switching from exon 9 to exon 8. First, we examined and compared the expression levels of these RNA-binding proteins between AT-3 and DT-3 cells by RT-PCR. Fox2 was expressed in both cell lines at similar levels, whereas expression of Fox1 was undetectable (Figure 4C), and both ESRP1 and ESRP2 were specifically expressed in epithelial type DT-3 cell (Figure 4C). Considering the observation that both UGCAUG and ISE/ISS-3 are essential cie-elements for selecting exon 8 (Figure 4B), broadly expressed Fox2 might cooperates with epithelial-specific ESRP1 and ESRP2 for exon 8 inclusion. To test this hypothesis, we transfected our FGFR2 splicing reporter with Fox1, Fox2, ESRP1, or ESRP2, or combinations of these into HeLa cell, which has non-epithelial cell character (Figure 5A, lane 1).

When Fox1 or Fox2 was introduced into HeLa cell, selection of exon 8 increased in a dose-depend manner and reached 10% (Figure 5A, lanes 2–4) or 40% (Figure 5A, lanes 5–7), respectively. When ESRP1 or ESRP2 was introduced, selection of exon 8 also increased in a dose-depend manner, and reached over 90% (Figure 5A, lanes 8–10 and 15–17, respectively). As HeLa cell expresses endogenous Fox2 in a similar manner to AT-3 cell (Figure 5A, lane 1), we introduced ESRP1 or ESRP2 under the Fox2 knockdown condition, and evaluated the cooperative effects of ESRP1 or ESRP2 with endogenous Fox2. The knockdown efficiency of Fox2 was more than 80% at the mRNA level (average 81.2%). Knockdown of endogenous Fox2 decreased the ratio of exon 8 selection promoted by ESRP1 or ESRP2, with a maximum reduction of 24% (35.1–11.0% Figure 5A, lane 8 versus lane 11) or 23% (55.9–32.6%, lane 15 versus lane 18), respectively. When both Fox2 and ESRP1 were transfected, the ratios of exon 8 inclusion were similar to those obtained with a single transfection of ESRP1 (Figure 5A, lanes 21–24). These results indicate that introduced ESRPs promote exon 8 inclusion with endogenous Fox2, suggesting that Fox2 and ESRPs cooperatively act together for exon 8 inclusion. These results were also confirmed by means of the fluorescence from the splicing reporter co-transfected with Fox1, Fox2, ESRP1, or ESRP2, or both Fox2 and ESRP1 (Figure 5B). Over-expression of ESRP1 or ESRP2 changed the color from red to green, but Fox1 or Fox2 alone had a smaller effect on the color change. Co-transfection of Fox2 and ESRP1 gave the maximum effect on the color switching which reflected the switching of proteins coded by the mutually exclusive exons. We then tested whether exon switching by Fox2 and ESRP1 depends on UGCAUG and ISE/ISS-3 in intron 8.

**Figure 6:**
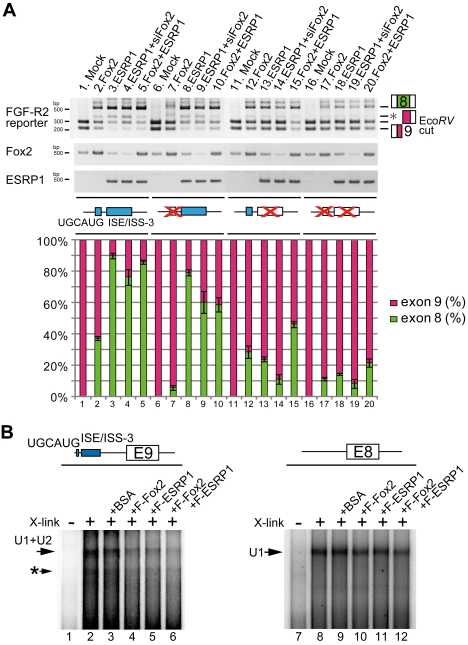
Foxs and ESRPs promote switching to exon 8 through repressing exon 9 via UGCAUG and ISE/ISS-3. (A) RT-PCR from HeLa cell transfected the indicated wild-type or cis-mutated reporter vectors and Fox2 and/or ESRP1 expression vectors with or without Fox2 siRNA. The bar graph shows the amount of each splicing product, and is based on calculations from three independent experiments; the mean value for each splice product is show in the respective column, with an error bar showing the SD (standard error). (B) Result of an in vitro splice site recognition assay. The scheme for exon 9 RNA probe shows the position of UGCAUG and ISE/ISS-3. The asterisk shows the probe crosslinked with U1, which binds to the cryptic 5' splice site inside exon 9.

We performed overexpression study of Fox2 and/or ESRP1 on reporter vectors mutated on either UGCAUG, ISE/ISS-3, or both of them with or without knock down condition of endogenous Fox2. When Fox2 was transfected with the UGCAUG site-mutated reporter, the promoting effect of Fox2 on exon 8 inclusion was lost (Figure 6A, lane 7). Overexpression of ESRP1 still promoted exon switching of the UGCAUG-mutated reporter, but the ratio of exon 8 selection was slightly reduced (Figure 6A, lane 8) in comparison with the wild-type reporter (Figure 6A, lane 3). To the contrary, when ISE/ISS-3 was mutated, the promotion effect of ESRP1 on the exon switching was significantly reduced (Figure 6A, lanes 13), and the effect of Fox2 remained (Figure 6A, lanes 12 and 15). When both UGCAUG and ISE/ISS-3 were mutated, neither Fox2 nor ESRP1 caused a drastic switching any more (Figure 6A, lane 16–20). These results showed that Fox and ESRP cooperatively promote switching from exon 8 to 9 through the cis-elements of UGCAUG and ISE/ISS-3 located near exon 9.

Next, we tested whether Fox and/or ESRP cause switching from exon 9 to exon 8 through interruption of exon 9 recognition. We examined this under the in vitro splicing conditions using ^32^P-labeled RNA probe of exon 9 with introns containing UGCAUG and ISE/ISS-3 sites (Figure 6B, top panel) and the exon 8 RNA probe of same stretches. When the exon 9 probe was crosslinked by UV irradiation after incubation with HeLa nuclear extract and separated by electrophoresis, shifted band by closslinking U1 and U2 was observed as overlap (Figure 6B, lane 2, indicated by arrow), as same as Figure 3C. However, in the exon 8 probe, only shifted band by U1 was observed (Figure 6B, lane 8, indicated by arrow, and data not shown for RNaseH digestion), presumably due to its weaker 3′ splice site. When recombinant Fox2 or ESRP1 protein was added with the exon 9 probe, shifted binds by U1 and U2 snRNA were decreased (Figure 6B, lanes 4 and 5, respectively), and almost disappeared by addition of both Fox2 and ESRP1 proteins (Figure 6B, lane 6). However, suppressive or activating effect of Fox2 or/and ESRP1 was not obvious with exon 8 probe (Figure 6B, lanes 9–12). These data indicate that both Fox and ESRP interrupt exon 9 recognition in vitro. Combining results from Figure 5 and Figure 6 indicate that Fox and ESRP disrupt exon 9 recognition from its 3′ splice site through UGCAUG and ISE/ISS-3, and promoted switching to exon 8.

### Expression profile of Fox and ESRP coincide with exon8-EGFP *in vivo*

**Figure 7:**
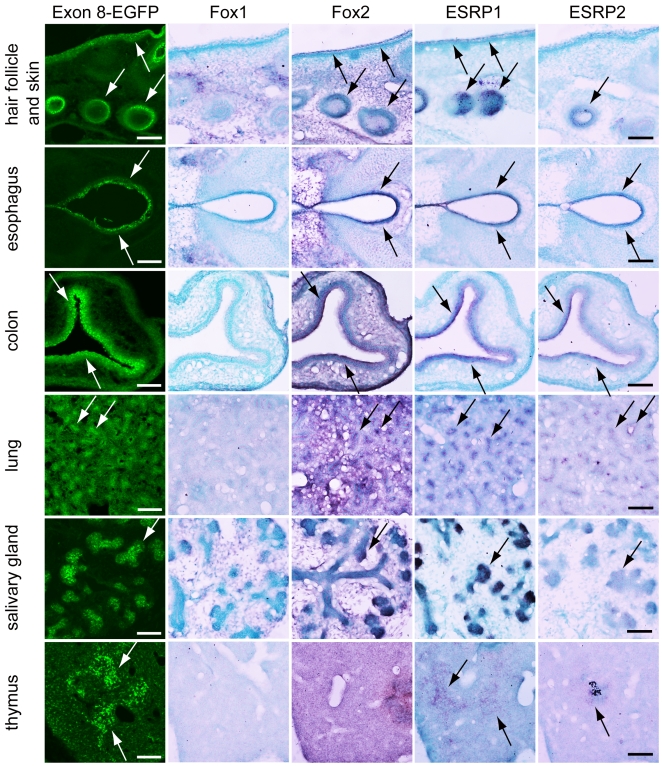
Expression pattern of Foxs and ESRPs in splicing reporter mouse embryos. Sections from transgenic reporter embryos at E16.5. EGFP signal showed the exon 8 splicing pattern, and in situ hybridization was performed with indicated probes using serial sections. The EGFP signal is indicated by a white arrow. The violet signal, indicating mRNA localization, is shown by arrows, and nuclei were counterstained with Methyl Green (scale bar = 100 µm).

**Figure 8:**
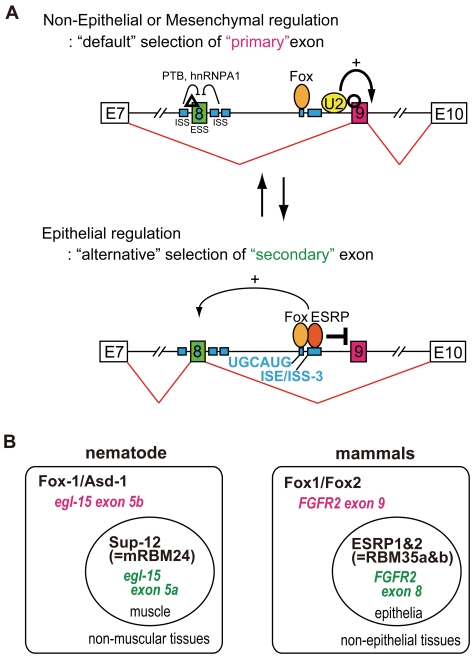
Model for tissue-specific splicing regulation of FGFR2 gene. (A) “*The Knight's Fork*” regulation model: In non-epithelial or mesenchymal tissues, exon 9 is chosen as “primary” exon due to its stronger 3′ splice site than exon 8 (“default” selection to choose “primary” exon). In epithelial tissues, “key regulators” repress exon 9 utilizing its 3′ splice site dependency for exon recognition and cause switch to “secondary” exons (“alternative” selection to choose “secondary” exon). A small number of “key regulators” can control two mutually exclusive exons through modifying ordered splice-site recognitions in a tissue-specific manner, resembling the way that a chess piece can simultaneously attack a rook and check the king. (B) In nematode, mutually exclusive splicing of the worm FGFR homologue gene of *egl-15* is regulated by the cooperation of broadly expressed Fox-1/ASD-1 family and the muscle-specific RNA binding motif protein (RBMs) of SUP-12 (a worm homologue of mRBM24), which act together to repress inclusion of alternative exon 5B to promote muscle-specific expression of exon 5A. In the case of mammalian FGFR2, the Fox family cooperates with the tissue-specific factor ESRPs (RBM35a and b) to repress alternative inclusion of exon 9 and to promote epithelial tissue-specific expression of exon 8.

In vitro study showed that disruption of exon 9 recognition from its 3′ splice site by Fox and ESRP through UGCAUG and ISE/ISS-3 promoted switching to exon 8. Remaining question is whether the expression of Fox and ESRP coincides with the expression of exon 8 form in tissue-specific manner during development in vivo. We examined the expression profiles of Fox1, Fox2, ESRP1, and ESRP2 by in situ hybridization using the serial sections from our reporter transgenic mice embryos at E16.5 (Figure 7). As we have already shown in Figure 1D, the exon 8-EGFP expression was on left panels (as indicated by white arrows). In the in situ hybridization performed with adjacent sections, Fox1 expression was not detected in tissues where the EGFP signal was observed, whereas strong signal of Fox1 was detected mainly in neuronal tissues in the same sections (data not shown). To the contrary, Fox2 mRNA was detected broadly throughout whole embryos at this stage, and its expression was overlapped with exon 8-EGFP signals localized in the epithelial tissues (indicated by black arrows). The expression of ESRP1 and ESRP2 was specifically detected in epithelial tissues (indicated by black arrows) and these expression almost completely overlapped with exon 8-EGFP signals during developing stage. These observations in vivo support an epithelial regulation model in Figure 8A in which tissue specific factor ESRPs act together with generally expressed Fox family to promote exon 8 inclusion.

## Discussion

Several groups have generated reporter system to visualize splicing regulation of FGFR2 gene. Newman et al. generated fluorescent reporter vectors with the minimum genomic region that can reflect endogenous splicing regulation in AT-3 and DT-3 cells. With this reporter, they identified specific ISEs, including ISS/ISS-3, that respond to Fox regulation [31]. Bonano et al. constructed another fluorescent reporter vector by using the genomic fragment around exon 8, and they visualized the regulation of exon 8 inclusion in the mouse [32]. In this study, we originally developed a transgenic reporter system using FGFR2 gene as model and succeeded in visualizing tissue-specific expression profiles of the mutually exclusive exons through differential expression of EGFP and RFP in mice for the first time to our knowledge. Using the mostly entire genomic region around alternative exons with their upper and lower constitutive exons, we could evaluate which splice site sequences and cis-elements are truly critical for regulations. This system has great advantage, 1) to monitor the dynamism of splicing regulation in vivo with single cell resolution, 2) to identify essential cis-elements and trans-factors, and reveal the hidden mechanisms of splicing regulation like this study, 3) to identify the essential candidate factors by fluorescent color change or to screen the regulators using cDNA or siRNA library. In this way, splicing reporter system has great advantage to decipher the hidden splice code and also to reveal the important roles regulated by alternative splicing during development or in adult with tissue- or cell-type specific manner.

With splicing reporter system, we showed that two mutually exclusive exons of FGFR2 gene seem to have “primary” and “secondary” fates. Without tissue-specific regulators, “primary” exon 9 is chosen as the “default” and the “secondary” exon 8 is silenced due to its weaker 3' splice site with exonic and intronic splice silencers sequence around exon 8 (Figure 8A, Non-Epithelial or Mesenchymal regulation). When tissue-specific key regulator ESRPs are expressed, these factors bind close to the 3' splice site of “primary” exon 9 with Fox, repress exon 9 recognition, and then “secondary” exon 8 will be chosen instead of exon 9 (Figure 8A, Epithelial regulation). In this step, exon 8 selection may be activated by ESRPs and/or Foxs because ESRPs are cloned as activators of 5′ exon when its binding site is located in downstream intron [16], [30], [31]. Our observation that overexpression of ESRPs mostly caused switching from exon 8 to exon 9, but did not increase the double-skip form (Figure 5), may be in good accordance with this hypothesis. However, in our X-link experiment with RNA probe possessing both exon 8 and UGCAUG+ISE/ISS-3 on its down-stream intron (intron 7, 77 bp; exon 8, 148 bp; intron 8,122 bp; UGCAUG+ISE/ISS-3 portion from 5′ of exon 9, 165 bp), we could not observe the increment of shifted band by addition of Fox2 and ESRP1 (data not shown). The deleted elements (ISE2, ISAR, and so on) or RNA structure may also be involved in the ESRPs-dependent promotion of exon 8 recognition, although more clarification is required.

Mutually exclusive alternative splicing regulated through the ordered splicing recognition with tissue specific factors has been observed in nematodes [33]. With this ordered splice-site recognition, transacting regulators can sequentially control the selection of mutually exclusive exons in a tissue-specific manner, in a similar manner to which a chess piece, usually a knight, can simultaneously attack a rook and check the king so that the former must be lost (Figure 8A). This regulatory mechanism clearly answered to our initial questions described in introduction. So far, tissue-specific alternative splicing regulation has been understood from the viewpoint of the balance between enhancement and silencing of the alternative exons mediated by trans-factors through specific cis-elements [3]. In this idea, each alternative exon is regulated discretely, and the final splicing isoforms are determined from the sum and balance of these independent regulations. If this were the case in mutually exclusive alternative splicing, the initial splicing products would be mixtures of the single-exon-inclusion form, double-inclusion form, and double-skip form. As the last two isoforms would be eliminated by NMD [25], only the single-exon-inclusion forms would remain as final splicing products. On the other hand, if mutually exclusive splicing is regulated by the switch-like manner, the initial spliced products would consist mainly of one of the single-inclusion forms. If the exon selection in AT-3 cell is on a balance of combined regulations of enhancer for exon 9 and repressor for exon 8, overexpression of repressor for exon 9 should produce mostly the double-skip form. We did not, however, observe this (Figure 5A). In addition, when the 3' slice site of exon 8 in the reporter was changed to be as strong as that of exon 9, the double-inclusion splicing product became dominant in AT-3 cell, without any change in the transacting factors or specific cis-elements affecting exon 8 (Figure 2, lane 2). Also, splice site mutation of exon 9 caused switching to epithelial exon 8 in non-epithelial AT-3 cell (Figure 3, lane 2 and 4). These results suggest that alternative splicing is not achieved by a complicated balance of multiple regulators, but is defined by a simple switch-like mechanism in which only one appropriate exon can be selected and switched by key regulators such as ESRPs.

Our previous study showed that mutually exclusive splicing of the worm FGFR homologue gene *egl-15* is regulated by the cooperation of broadly expressed Fox-1/ASD-1 family and the muscle-specific RNA binding motif proteins (RBMs) of SUP-12 (a nematode homologue of mRBM24), which act together to repress inclusion of alternative exon 5B to promote muscle-specific expression of exon 5A [34], [35] (Figure 8B, nematode). In the case of mammalian FGFR2, the generally expressed Fox family cooperate with the tissue-specific factor ESRPs (RBM35a & b) to repress alternative inclusion of exon 9 and to promote epithelial tissue-specific expression of exon 8 (Figure 8B, mammals). Moreover, both of nematode and mammalian Fox family proteins bind to the UGCAUG element, and SUP-12 and ESRPs bind to GU stretches. Nematode has rather similar number of genes (∼20,000) comparing with mammals but the size of its genome is much smaller (about 1/30) with very shorter intron (average 561 bp). So, alternative splicing in nematode is considered to be regulated with much simpler rules [36], [37]. Thus, it is amazing that regulatory mechanism of tissue-specific alternative splicing is evolutionally conserved from nematode to mammal, and regulation though Fox and another tissue-specific RBMs may turn out to be a widespread essential phenomenon to regulate tissue-specific alternative splicing of multiple genes. Our findings also provide the significant cue to understand how a number of spliced genes are regulated in various tissue-specific manners by a limited number of regulators, eventually to understand seemingly complicated developmental process or tissue-specific functions.

## Materials and Methods

### Plasmid construction

We constructed the reporter vector essentially as described in the text. The FGFR2 genomic region spanning from exon 7 to exon 10 from mouse genomic DNA was amplified by PCR and cloned into Gateway Destination vector (Invitrogen), carrying both EGFP (Clontech) and mRFP [38] with different reading frames, under the control of the CAGGS promoter. To stabilize and enhance reporter protein expression, artificial sequence of modified glutathione S-transferase (GST) gene (QIAGEN) was introduced in front of the FGFR2 genomic fragment and connected in frame with exon 7. A 1-kbp fragment at the center of the FGFR2 intron 9 was removed because of its significant reducing effect in mRNA expression. Mutations of cis-elements were introduced by using QuikChange XL II (Stratagene). Mouse Fox1 and Fox2 cDNAs were kindly provided by Dr. Kawamoto [39] and cloned into the Gateway Destination vector driven by a CAGGS promoter. Primer sequences for amplifying FGFR2, deleting 1 kbp in the middle of intron 9, and introducing mutations were indicated in Methods S1.

### Generation of FGFR2 splicing reporter mice

The constructed FGFR2 splicing reporter vector was linearized and injected into the pronucleus of a C57BL/6 oocyte. Mice were genotyped by PCR with primers for EGFP using genomic DNA from the postnatal and late embryonic tails, or yolk sacs from earlier embryos.

### Ethics statement

All experiments involving animals were performed in accordance with the protocols certified by the Institutional Animal Care and Use Committee of the Tokyo Medical and Dental University (Approval Numbers #0070220, #0080179, and #0090084).

### Cell culture and transfection

Rat AT-3 and DT-3 prostate carcinoma cell lines were kindly provided by Dr. Garcia-Blanco and Dr. Carstein. Cells were maintained in Dulbecco's modified eagle medium (D-MEM) with 10% fetal calf serum (FCS), and vectors were transfected with TransFectin (BioRad). Stealth siRNA was used in knockdown experiments on endogenous Fox2 and was transfected using Lipofectamine RNAiMAX (Invitrogen).

### Microscopy

Fluorescent images of whole embryos of reporter transgenic mice were capture under a fluorescence microscope (MZ16FA, Lecia) with a charge-coupled device (CCD) camera (DP71, Olympus). We also used a confocal microscope (Fluoview FV1000, Olympus) to capture fluorescent images of sectioned transgenic embryos, and the captured images were processed by means of Metamorph (Molecular Devices). Fluorescent images of cultured cells and bright-field images showing in situ hybridization were captured by using a Nikon Eclipse E600 microscope with a CCD camera (DP71, Olympus).

### RT-PCR

Total RNAs and RT-PCR were performed as described previously [34], [40]. The identity of all splicing variants was confirmed by sequencing. Amounts of PCR products were measured with a 2100 BioAnalyzer with Agilent DNA1000 kits (Agilent Technology), and the quantitative analyses were performed in more than three independent experiments. Primer sequences used in the RT-PCR assays are listed in Methods S2.

### *In vitro* exon-recognition assay

The PCR products of T7-ex 9 wt (mouse FGFR2 intron 8, 200 nt; exon 9, 145 nt; and intron 9, 105 nt), T7-ex 9 containing UGCAUG and ISE/ISS-3 in intron 8 (intron 8, 237 nt; exon 9, 145 nt; and intron 9, 74 nt), and T7-ex 8 wt (intron 8, 200 nt; exon 8, 149 nt; and intron 9, 100 nt) were used as DNA templates for T7 transcription. A mutated DNA template of 3' ss in intron 8 and 5' ss in intron 9 was prepared from mutated reporter vectors as shown in Figure 3A. The RNA substrates were labeled with ^32^P by in vitro T7 transcription. In vitro splicing reaction and UV cross linking were performed under the conditions described by Sawa [29]. To identify the shifted bands by UV cross linking, pre-heated cDNA oligo (10 µg/mL) for U1 (5'-CGGAGTGCAATG-3') or U2 (5'-CAGATACTACACTTG-3') was added to the RNAs after UV cross linking, and the U1 or U2 cDNA oligo/RNAs mixture were digested with 50 U/mL of RNase H at 30°C for 10 min. In Figure 6A, highly purified Flag-Fox2 and Flag-ESRP1 were added to RNAs and incubated at room temperature for 5 min before splicing reaction to examine their inhibitory or activating effects on exon recognition of U1 snRNA or U2 snRNA. After these reactions, RNAs were subjected to denaturing PAGE analysis and autoradiography.

### *In situ* hybridization

In situ hybridization was carried out as previously described [41], [42], with modifications. Briefly, embryos were fixed with 4% paraformaldehyde, cryo-protected with 30% sucrose, embedded in optimal cutting temperature (OCT) compound, and cut into sections in 20 µm thickness. Antisense RNA probes labeled with digoxigenin were visualized with Fab fragments from an antibody against digoxigenein conjugated with alkaline phosphatase (Roche) and 5-bromo-4-chloro-3-indolyl phosphate (BCIP)/Nitroblue Tetrazolium (NBT) solutions. Sections were counterstained with Methyl Green. The following cDNA was used as the riboprobe: *Fox1* (131–865 bp from mouse cDNA), *Fox2* (2261–2991 bp from mouse cDNA), *ESRP1* (1168–1708 bp from mouse cDNA), and *ESRP2* (208–807 bp from mouse cDNA).

## Supporting Information

Figure S1 In vitro splice site recognition assay of exon 9 RNA probe with snRNA oligos “X-link” shows the presence or absence of UV-induced crosslinks in samples after the in vitro splicing reaction. U1, U2, U2(2), U2(3), U1+U2, or U6 oligos represent the digestion of RNA samples by RNaseH with complementary oligos, respectively. U2 complementary oligos of U2(2) and U2(3), which target different portion of U2 snRNA, were used to compare the digestion efficiency. Shifted band almost disappeared with double digestion using U1+U2 oligos (lane 7), while the band was resistant against the digestion with U6 oligo (lane8), indicating that the exon 9 RNA probe is recognized by U1 and U2 snRNA. The band shown by arrowheads with asterisk may be a probe crosslinked with U1 that binds to the cryptic 5' splice site inside exon 9, as already described in the figure legend of Fig-3C. Sequence of U2(2) and U2(3) oligos U2: same oligo used in Fig-3 U2(2): cagtttaatatctg U2(3): ccatttaatatatt U6: cgcttcacgaatttgcgt.
(5.24 MB TIF)

Methods S1 Primer sequences for amplifying FGFR2, deleting 1 kbp in the middle of intron 9, and introducing mutations.
(0.02 MB DOC)

Methods S2 Primer sequences used in the RT-PCR assays.
(0.02 MB DOC)
