# Supplementary figures and images for: Splicing Reporter Mice Revealed the Evolutionally Conserved Switching Mechanism of Tissue-Specific Alternative Exon Selection
Source: PLoS One. 2010 Jun 3;5(6):e10946. doi: 10.1371/journal.pone.0010946 (PMC2880598; doi:10.1371/journal.pone.0010946)

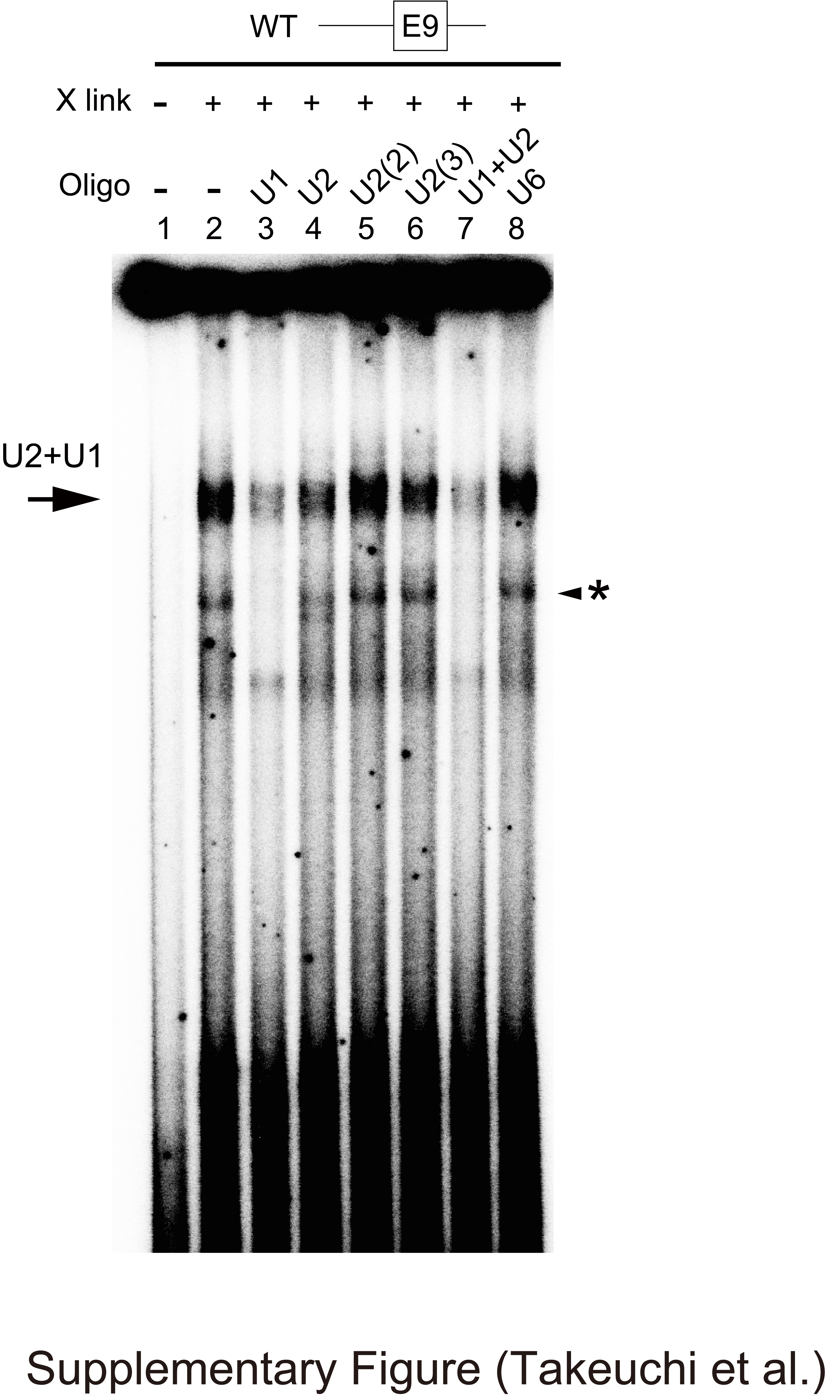

Supplement: Figure S1 — In vitro splice site recognition assay of exon 9 RNA probe with snRNA oligos “X-link” shows the presence or absence of UV-induced crosslinks in samples after the in vitro splicing reaction. U1, U2, U2(2), U2(3), U1+U2, or U6 oligos represent the digestion of RNA samples by RNaseH with complementary oligos, respectively. U2 complementary oligos of U2(2) and U2(3), which target different portion of U2 snRNA, were used to compare the digestion efficiency. Shifted band almost disappeared with double digestion using U1+U2 oligos (lane 7), while the band was resistant against the digestion with U6 oligo (lane8), indicating that the exon 9 RNA probe is recognized by U1 and U2 snRNA. The band shown by arrowheads with asterisk may be a probe crosslinked with U1 that binds to the cryptic 5' splice site inside exon 9, as already described in the figure legend of Fig-3C. Sequence of U2(2) and U2(3) oligos U2: same oligo used in Fig-3 U2(2): cagtttaatatctg U2(3): ccatttaatatatt U6: cgcttcacgaatttgcgt. (5.24 MB TIF) [file pone.0010946.s001.tif]
