# Supplementary material for: Splicing Reporter Mice Revealed the Evolutionally Conserved Switching Mechanism of Tissue-Specific Alternative Exon Selection
Source: PLoS One. 2010 Jun 3;5(6):e10946. doi: 10.1371/journal.pone.0010946 (PMC2880598; doi:10.1371/journal.pone.0010946)
Supplement: Methods S1 — Primer sequences for amplifying FGFR2, deleting 1 kbp in the middle of intron 9, and introducing mutations. (0.02 MB DOC) [file pone.0010946.s002.doc]

FGFR2 amplify-F : ggctgccctacctcaaggtcctg

FGFR2 amplify-R : ctctctcacaggcgctgggttgcag

intron 9 partial deletion-F : gcatgccttgatagagtggcctctc-ctgttgaaccttcccctggag

intron 9 partial deletion-R : ctccaggggaaggttcaacag-gagaggccactctatcaaggcatgc

exon 9 mutation for deleting PTC-F: gaggttctctatattcggaatgtTacttttgaggatgctggg

exon 9 mutation for deleting PTC-R: cccagcatcctcaaaagtAacattccgaatatagagaacctc

exon 9 3’ splice site mutation-F : gcttcgtttgttttctctgccgccggtgttaacaccacggac

exon 9 3’ splice site mutation-R : gtccgtggtgttaacaccggcggcagagaaaacaaacgaagc

exon 9 5’ splice site mutation-F : ctgcatggttgacagttctgccaccaacatactgctctttctctc

exon 9 5’ splice site mutation-R : gagagaaagagcagtatgttggtggcagaactgtcaaccatgcag

TGCATG mutation -F : gggccaatttttccatgtgttcaatttacgtacgttctaggtggtgacg

TGCATG mutation -R : cgtcaccacctagaacgtacgtaaattgaacacatggaaaaattggccc

ISE/ISS-3 mutation -F : taggtggtgacgccgaatctcctgatggcc

ISE/ISS-3 mutation -R : ggccatcaggagattcggcgtcaccaccta
