# Supplementary material for: Splicing Reporter Mice Revealed the Evolutionally Conserved Switching Mechanism of Tissue-Specific Alternative Exon Selection
Source: PLoS One. 2010 Jun 3;5(6):e10946. doi: 10.1371/journal.pone.0010946 (PMC2880598; doi:10.1371/journal.pone.0010946)
Supplement: Methods S2 — Primer sequences used in the RT-PCR assays. (0.02 MB DOC) [file pone.0010946.s003.doc]

Reporter FGFR2-F 5'-GGCCTTTGCAGGGCTGGC-3'

Reporter FGFR2-R 5'-GGAGCCGTACTGGAACTGAGG-3'

Fox1-F 5'-GGCACCGCCACACAGACAGATGA-3'

Fox1-R 5'-TCCTGGTTGGCCTGGCACAACAG-3'

Fox2-F 5'-CAACAACTCCTGACGCAATGGTTCAGC-3'

Fox2-R 5'-GATTTTACGGCCCTCTACCACGGTG-3'

ESRP1-F 5'-CAGGAGATGCCTTTATCCAGATGAAGTC-3'

ESRP1-R 5'-CAGTATTGTAGGCCAGGCCCTG-3'

ESRP2-F 5'-CCTACACAGCCACCATTGAAGACATTC-3'

ESRP2-R 5'-GGTGAGGTAGCCCACAGTAGTG-3'
